# Model Design and Study of a U-Channel Photonic Crystal Fib Optic Sensor for Measuring Glucose Concentration in Blood

**DOI:** 10.3390/s25092647

**Published:** 2025-04-22

**Authors:** Lei Zhao, Hua Yang, Tangyou Sun, Qianju Song, Zao Yi, Yougen Yi

**Affiliations:** 1 Joint Laboratory for Extreme Conditions Matter Properties, The State Key Laboratory of Environment-Friendly Energy Materials, School of Mathematics and Science, Tianfu Institute of Research and Innovation, Southwest University of Science and Technology, Mianyang 621010, China; 18383235873@163.com (L.Z.); qjsong@swust.edu.cn (Q.S.); 2 School of Science, Lanzhou University of Technology, Lanzhou 730050, China; hyang@lut.edu.cn; 3 Guangxi Key Laboratory of Precision Navigation Technology and Application, Guilin University of Electronic Technology, Guilin 541004, China; suntangyou@guet.edu.cn; 4 School of Chemistry and Chemical Engineering, Jishou University, Jishou 416000, China; 5 College of Physics, Central South University, Changsha 410083, China

**Keywords:** surface plasmon resonance, u-channel photonic crystal fiber, biosensing, glucose concentration detection in blood

## Abstract

This research introduces a biosensor utilizing surface plasmon resonance in a photonic crystal fiber (PCF) configuration. PCF uses fused silica as the base material, with a layer of gold placed over the U-channels in the cross-section of the fiber to create a surface plasmon resonance. There are three different sizes of internal fiber optic air hole diameters, with a larger channel circle below the u-channel for the formation of an energy leakage window. COMSOL software 6.0 assisted us in tuning the fiber optic structure and performance for the study, and the structural parameters analyzed mainly include the channel circle diameter, the channel circle spacing, the profundity measurement of the polished layer, and the nanoscale size variation of metal films. The results of the simulation study show that the optical fiber sensor achieves refractive index (RI) responsiveness across the 1.30 to 1.41 range, and in the RI interval of 1.40 to 1.41, the sensor exhibits the largest resonance peak shift, and its highest sensitivity reaches 10,200 nm/RIU, and the smallest full width at half peak (FWHM) corresponds to the RI of 1.34 with a value of 4.8 nm, and the highest figure of merit (FOM) corresponds to the RI of 1.34 with a value of 895.83 (1/RIU). COMSOL 6.0 simulation software, was used to simulate the changes in blood refractive index corresponding to different glucose concentrations, and the detection performance of the sensor for different concentrations of glucose was tested. Then, the results show that the glucose concentration in 75 mg/dL–175 mg/dL with RI detection sensitivity is 3750 nm/RIU, where the maximum refractive index sensitivity is 5455 nm/RIU. It shows that the sensor can be applied in the field of biomedical applications, with its convenience, fast response, and high sensitivity, it has great potential and development prospect in the market.

## 1. Introduction

In today’s technological era, sensors found extensive utilization across multiple domains, encompassing industrial automation and control, environmental monitoring, medical diagnostics and therapy, automotive and transportation, smartphones and wearables, agriculture and food safety, security monitoring, and Internet of Things applications [1,2,3,4]. For example, photonic crystal fiber sensors with surface plasmon resonance enhancement have the advantages of flexible design, low-loss transmission, high sensitivity, high nonlinearity, miniaturization and integration, a wide range of applications, real-time monitoring, etc. [5,6]. Fano proposed the theory of surface plasmon resonance in 1941 [7], and in 1966, K.C. Kao, Hockham, and Werts, based on the dielectric waveguide theory proposed the concept of the optical fiber transmission line [8], and the world’s first optical fiber was successfully developed in 1970, marking the beginning of the era of optical fiber communication. After 1977, people began to carry out research on optical fiber sensing technology to achieve the effective combination of optical fiber and surface plasmon resonance theory. Due to the complex material parameters and structural variability of photonic crystal fiber (PCF)-based SPR sensors [9], we employ computer simulations and the finite element method (FEM) to analyze the electromagnetic field distribution in the PCF-SPR structure. Next, it is necessary to understand the basic theory of PCF-SPR. Surface plasmon resonance (SPR) arises from the optical excitation of surface plasmon polaritons (SPPs), a resonant electromagnetic phenomenon resulting from the coupling between incident photons and collective electron oscillations at the dielectric–metal interface [10,11,12]. Light incident upon the surface of the dielectric undergoes total reflection, and the resulting evanescent wave stimulates the free electrons located on the metal surface, thus giving rise to the generation of the surface plasmon wave (SPW). The surface plasmon resonance phenomenon takes place while the frequency belonging to the swift wave is the same as that of the SPW [13]. A large proportion of the energy of the incident light is taken in by the surface plasmon wave when the surface plasmon resonance phenomenon takes place. This triggers the energy transfer from photons to surface plasmons. Consequently, the intensity of the reflected light is substantially reduced, and a resonance absorption peak appears in the outgoing spectrum [14,15]. Surface plasmon resonance exhibits high sensitivity to alterations in the index of refraction near the metal surface. A change in the refractive index causes a corresponding alteration regarding the strength of the resonant peaks. This characteristic is the reason why it is extensively employed in the field of refractive index sensing [16,17,18]. Photonic crystal fibers, which are also referred to as microfibers, are generally made of silica glass as the basic skeleton, and the cross-section features air holes that are arranged periodically. These air holes run longitudinally along the fiber, forming a photonic crystal structure that can be designed to modulate the propagation characteristics of light through its geometry. Due to its unique internal structure of multiple air channels, it not only transmits light, but also serves as a sensing channel for the interaction of light with the biological substance to be measured, which is a unique advantage for use in biosensors. Compared with traditional optical fibers, photonic crystal fibers have the advantages of portability, low loss, high nonlinearity, birefringence effect, and wireless single mode [19,20]. PCFs are classified into two types: total internal reflection photonic crystal fibers and photonic band gap photonic crystal fibers. For the former, the basic principle of light conduction is that the refractive index of the optical fiber cladding consisting of an air hole arrangement in the PCF that is lower than the core’s refractive index, and the light always tends to the substance of the high refractive index so that the light wave can be bound in the core layer. Additionally, the latter relies on the photonics band gap (photonics band gap) effect to conduct light waves [21].

Traditional SPR sensors are usually based on the rationale of attenuation of total reflection, using prisms or planar waveguide structures, which have high sensitivity but are large, costly, and difficult to miniaturize and integrate [22,23]. With major breakthroughs in photonic crystal fiber design and preparation processes, researchers successfully integrated the surface plasmon resonance effect with the PCF structure to develop a series of new optical sensors based on the PCF-SPR coupling mechanism for applications in different fields [24]. SPR technology emerged as a trendy topic in the biosensing field owing to its advantages of real-time detection capability, high sensitivity, and no need for labeling. In the field of biosensing, different analytes have different refractive indices, and different concentrations of the same analyte have corresponding refractive indices, so biosensors based on RI changes can be developed according to this theory to analyze biological fluids and achieve rapid detection at the same time [25,26]. Glucose concentration in blood is an important physiological indicator of the metabolic state of the human body, and its normal reference range is usually 80–120 mg/dL during fasting [27]. Prolonged hyperglycemia may lead to various complications, such as cardiovascular diseases, renal lesions, retinopathy and neuropathy, etc. Hypoglycemia may lead to acute symptoms, such as coma, which require emergency treatment [28], so regular testing of glucose concentration in blood can be used for monitoring health conditions.

In this study, the proposed PCF-SPR biosensor can be used to measure glucose concentration in blood and other biological fluids in the corresponding refractive index range. The optical fiber sensor proposed in the article is a U-channel photonic crystal fiber with a layer of Au film coated on the U-channel. The U-channel decreases the gold film coating thickness to reduce the manufacturing cost and simplify the process, and it also significantly improves the contact area of the light field with the substance to be measured, enhances the swift field effect, and improves the sensing sensitivity. Gold has excellent surface plasmonic activity, biocompatibility, stability, and a strong SPR effect within the visible light and near-infrared spectral ranges, enabling it to detect analytes (e.g., glucose, proteins, DNA, etc.) at very low concentrations [29,30]. Below the U-shaped channel of the biosensor are two larger channel circles for forming an energy leakage window. The fiber core is surrounded by the smallest diameter air holes and the outer layer is a periodic row of normal air holes through the addition of a perfectly matched layer (PML) that captures radiant energy from the PCF surface through scattering boundary conditions. The structural parameters of the PCF are optimized using software simulation and numerical modeling to find out the optimum structural optimization through the control variable method. The final findings suggest that that the sensor response is in the interval of 1.30–1.41, and within the refractive index interval of 1.40 to 1.41, the sensor exhibits the largest resonance peak shift with a peak sensitivity of 10,200 nm/RIU, the smallest FWHM corresponds to an index of refraction of 1.34 with a value of 4.8 nm, and the highest FOM counterparts to a refractive index of 1.34 with a value of 895.83 (1/RIU). The sensor’s reaction at various concentrations was also examined using the determination of the glucose level in blood as an example, and the results show that the glucose concentration was in the range of 75 mg/dL–175 mg/dL, and sensitivity to refractive index was 3750 nm/RIU, where the highest refractive index sensitivity was 5455 nm/RIU.

## 2. Structural Parameters

**Figure 1:**
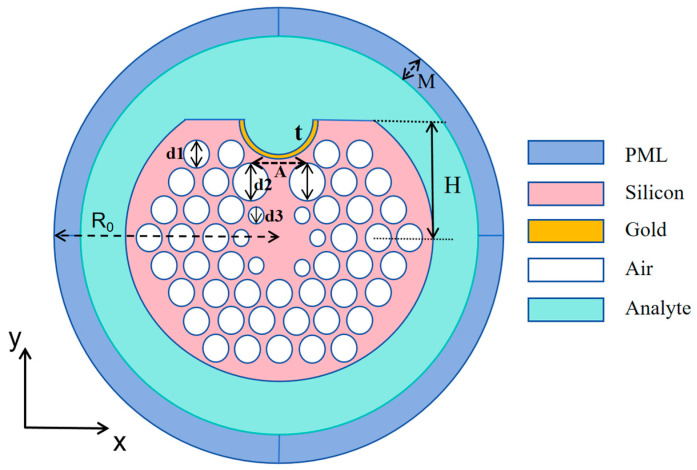
Model diagram of u-sensor in this paper.

Figure 1 presents the cross-sectional schematic of the U-channel photonic crystal fiber-sensing structure. PCFs can usually be fabricated by the process of stacking and pulling technology. The D-shaped PCF is realized by polishing one side of the circular photonic crystal fiber, while the U-channel structure can be fabricated utilizing either the wheel polishing technique or the V-groove edge polishing approach [31]. Then, the U-channel is covered with metal using a vacuum coating technique with controlled precision, i.e., one layer near the core is the gold nanolayer, and the second layer constitutes the analyte liquid, which serves as the target medium for measurement. Light will be confined within the PCF core by the cladding pores, generating an unstable field that interacts with the gold layer and thus excites surface plasmonic free electrons [32]. The liquid layer to be measured contains a sample of the analyte to be identified, and for the purpose of eliminating the reflective impacts of the simulation boundary and avoid simulation errors, a perfectly matched layer (PML) is used that takes in the radiant energy from the PCF surface through the scattering boundary conditions.

The diameter of the sensor’s optical fiber is 22 μm, the U-channel has a diameter D of 3.6 μm, the ordinary air holes have a spacing P of 1.6 μm, and the thickness of the metal film is t. d1, d2, and d3 are the diameters of the No. 1, No. 2, and No. 3 air holes, respectively; the No. 1 air holes are ordinary air holes, the No. 2 air holes are the circle of the channel, and the No. 3 air holes are surrounded around the fiber core. The specific parameters are presented in Table 1.

We used fused silica as a filler material to prepare PCFs with an air refractive index of 1. The refractive index–wavelength relationship can be described by the Sellmeier dispersion model with the following expression [33]:

(1) $$n^{2}=1+\frac{{0.691663\lambda }^{2}}{\lambda^{2}-{\left(0.0684043\right)}^{2}}+\frac{0.4079426\lambda^{2}}{\lambda^{2}-{\left(0.1162414\right)}^{2}}+\frac{0.897479\lambda^{2}}{\lambda^{2}-{\left(9.896161\right)}^{2}}.$$

The *n* denotes the index of refraction of quartz glass, and the incident wavelength *λ* is given in units of μm.

According to the Drude dispersion model under the electron gas approximation, one can express the dielectric function associated with the metallic material in the form of [34,35]:

(2) $$\varepsilon =\varepsilon_{\infty }-\frac{\omega_{p}^{2}}{\omega^{2}+i\gamma \omega }$$

where *ε_∞_* is the dielectric constant at high frequencies, usually 1 ≤ *ε_∞_* ≤ 10. *ω_p_* denotes the plasma frequency of the electron, *ω* denotes the angular frequency of the electromagnetic wave, and *γ* denotes the damping constant [36].

In this paper, the metallic material is chosen as gold with better surface plasmon resonance excitation, and the Drude–Lorentz dispersion model is utilized to represent the permittivity of the free electrons in the thin film gold layer as follows [37,38]:

(3) $$Ε\left(\omega \right)=\varepsilon_{\infty }-\frac{\omega_{D}^{2}}{\omega \left(\omega +i\gamma_{D}\right)}-\frac{\Delta \varepsilon \cdot \Omega_{L}}{\left(\omega^{2}-\Omega_{L}^{2}\right)+i\Gamma_{L}\omega }.$$

Here, *ε_∞_* equals 5.9673. It is ω that represents the circular frequency. The damping frequency *γ_D_* is 31.84π THz. ω*_D_* stands for the plasma frequency, with ω*_D_/2π* being 2113.6 THz. Ω*_L_* refers to the frequency of Lorentz oscillations, having a value of 650.07 THz, and Γ*_L_* is the bandwidth of the Lorentz oscillations, where Γ*_L_*/2*π* amounts to 104.86 THz.

In PCF-SPR sensors, two important factors for measuring the sensor’s performance are the full width of half peak (FWHM) and the spectral sensitivity. The FWHM is the loss spectral width corresponding to half of the absorption spectrum’s peak, and the smaller its value, the better the sensor’s ability to resist noise interference [39]. Adjustment of the relevant microstructural parameters causes a drift for the coupling wavelength, and the spectrum sensitivity is counted using the following formula [40]:

(4) $$S_{\lambda }=\frac{∆\lambda_{peak\;}}{∆n_{a}}$$

∆*λ_peak_* is the shift of the coupling wavelength in relation to the refractive index; $∆n_{a}$ is the change in refractive index of the liquid to be measured.

In addition, we discuss the sensor’s figure of merit (FOM) proposed in this paper [41]:

(5) $$FOM=\frac{S_{\lambda }}{FWHM}.$$

The presumed minimum spectrum resolution is ∆*λ_min_*. For changes in analyte RI, the sensor’s resolution is expressed as follows [42]:

(6) $$R=\frac{∆n_{a\cdot }\lambda_{min}}{△\lambda_{peak}}.$$

**Figure 2:**
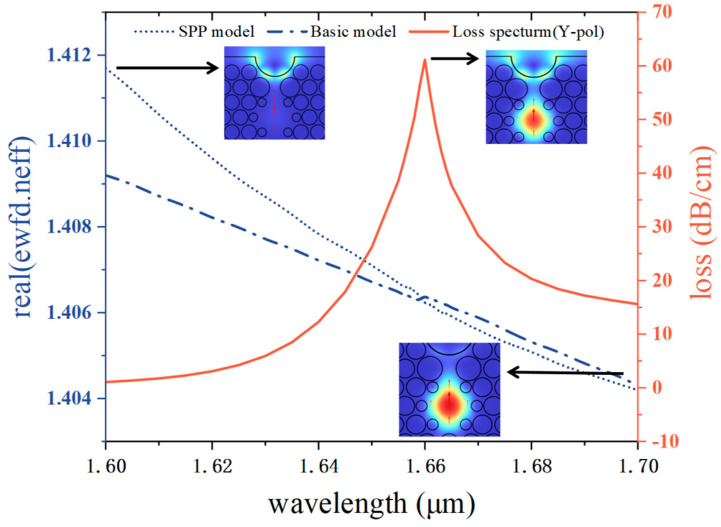
Dispersion curves and SPR loss-characteristic curves of Y-polarized fundamental and SPP modes for *n_a_* = 1.41. The inserted plots represent the electric field distribution in spp mode, fundamental mode, and at resonance, respectively, with the red arrows representing the orientation of the electric field.

The model proposed in this paper does not have a rotationally symmetric structure, and the impact of the attenuation profile of the X-polarized mode was found to be less excellent than that of the Y-polarized mode during the experiment, so the main discussion is on the loss of the y-polarized core mode during the propagation process [43]. As indicated in Figure 2, the SPR effect of this PCF coated with Au film is simulated and analyzed. In the refractive index of 1.41, the other parameters are set as t = 30 nm, d2 = 1.8 μm, d = 1.4 μm, and H = 5.6 μm. The red curve in the figure is the loss curve of the y-polarized direction of the y-pol core mode, and the resonance peaks of the y-pol state increase initially and subsequently decline along with the growth of the wavelength. The blue dashed–dotted line is the effective refraction index of the real part of the core mode, and the blue dotted line is the effective refractive index real part of the spp mode [44]. The effective index of the refraction real portion of both modes declines at varying speeds with the increase in wavelength. The analysis of the distribution of the electric field shows that the energy is moved from the fiber core to the surface of the Au layer with coupling strength in the y-pol paradigm in the 1.60 μm–1.70 μm band. From the dispersion curves and loss curves in Figure 2, it is obvious that the loss value of y-pol reaches the maximum while the real component of the effective index of refraction of the fundamental mode and the spp mode are intersected, indicating that the moment the energy transferred from the fiber core mode to the spp mode is the largest, so the spr effect is the strongest [45]. Presently, the coupling wavelength reaches 1.66 μm, with the peak loss value hitting 61.15 dB/cm.

## 3. Results and Analyses

The structure of PCF-SPR sensors is tiny, and smaller parameter adjustments can affect the SPR effect. Within the PCF-SPR sensor presented in the article, some of the structural parameters, such as the Au film thickness of the PCF, t, the channel circle diameter, d2, the channel circle spacing, d, and the polishing thickness, H, affect the refractive index distribution and mode coupling ability of the fiber, which in turn affects the sensor’s sensing properties. In this section, the above parameters are modeled and analyzed theoretically using the control variable method, and finally, the optimal structural parameters are selected to study the sensor refractive index detection range as well as the change in sensor performance due to the change in the refractive index of the analyte.

### 3.1. Au Film Thickness

**Figure 3:**
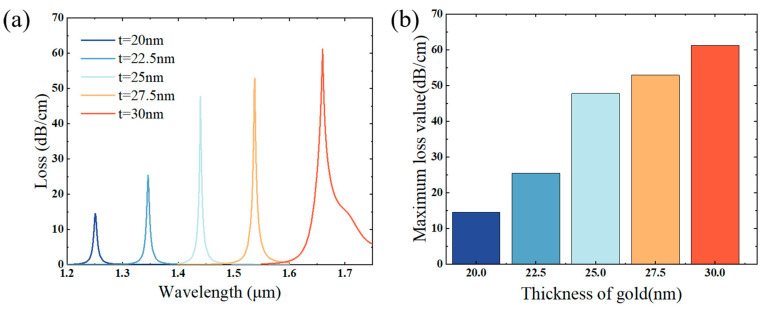
(**a**) The pattern of change in the loss spectrum with an index of refraction of 1.41 and gold layer thicknesses where t takes values of 20 nm, 22.5 nm, 25 nm, 27.5 nm, and 30 nm; (**b**) when nc = 1.38, the maximum loss value of the different thickness of the gold layer.

The surface plasma wave (SPW) shows great sensitivity to the alterations in the thickness of the metallic layer, the metal layer is the key to whether the SPR effect can be stimulated, and the effective adjustment of its thickness is the most important of the structure optimization [46,47]. If the gold film thickness exceeds the appropriate range, it will weaken the penetration ability of the SPW, resulting in significant damping loss, and the electric field will be unable to pass through the Au film, thus reducing the sensitivity and limiting the loss performance of the system, while in the case that the gold film depth is insufficient, it will strongly inhibit the excitation of the plasma excitations due to the radiative damping effect [48,49]. Therefore, the impact of the metal film depth t on the SPR effect at the u-channel is discussed firstly. Assuming that the liquid’s refractive index to be measured is 1.41, and keeping the other parameters unchanged, and taking 2.5 nm as a gradient, when the thicknesses of the gold nanofilms are 20 nm, 22.5 nm, 25 nm, 27.5 nm, and 30 nm, respectively, the loss spectra of the fundamental mode of the PCF on the basis of the SPR effect are illustrated in Figure 3a, and it is evident that when the Au film turns thicker, the SPR resonance wavelength is gradually shifted towards the red end of the spectrum, and the resonance wavelengths are 1.251 μm, 1.346 μm, 1.44 μm, 1.538 μm, and 1.66 μm, respectively, and the maximum loss values are 14.53 dB/cm, 25.38 dB/cm, 47.75 dB/cm, 52.87 dB/cm, and 61.15 dB/cm, and it is evident from observation that the loss value at 30 nm is obviously the highest. Considering the peak case and the structural constraints on the thickness of the Au film at the same time, a thickness of 30 nanometers for the gold (Au) layer is chosen.

### 3.2. Channel Circle Diameter

**Figure 4:**
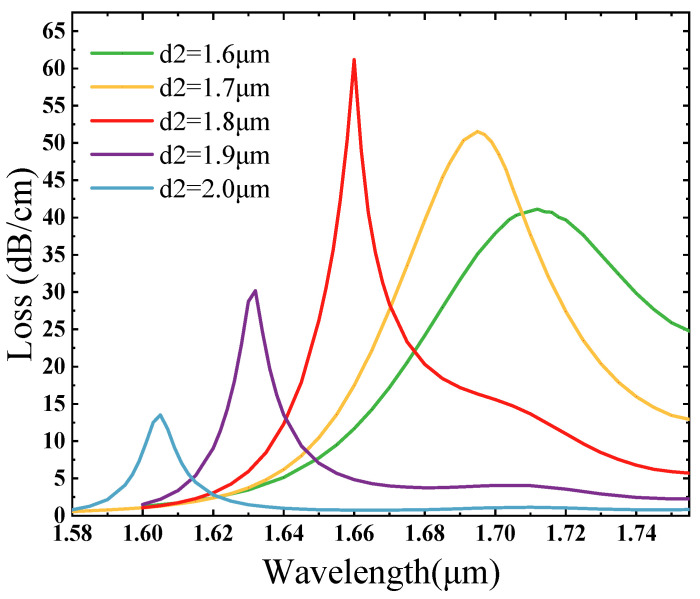
The change rule of the loss value when the refractive index is 1.41, the diameter of the channel circle d2 = 1.6 μm, d2 = 1.7 μm, d2 = 1.8 μm, d2 = 1.9 μm, and d2 = 2.0 μm.

Keeping other structural parameters unchanged, only the size of the channel circle diameter d2 was changed to investigate the impact of the variation in d2 regarding the loss spectrum. Figure 4 shows that in the case of the refraction index of the filled material to be measured being 1.41, with 0.1 μm as a gradient, the channel circle diameter rises from 1.6 μm to 2.0 μm, and it is evident that the resonance peaks increase and then decrease, and the resonant wavelengths are gradually blue shifted. This is because d2 increases so that the RI of the fundamental mode increases, and the SPP mode’s effective refractive index begins to decrease, so the coupling wavelength moves in the short wavelength region [50,51]. The coupling wavelengths are 1.712 μm, 1.695 μm, 1.66 μm, 1.632 μm, and 1.605 μm, respectively, and the loss increases from 41.12 dB/cm to 61.15 dB/cm and then reduces to 13.50 dB/cm. The reduction in the d2 size drives the excitation of stronger equipartitioned excitations at the open-loop channel, causing the shift in the loss spectrum towards longer wavelengths when a phase match is reached with the core mode. Too small a channel circle has weak constraints on the core, which may cause interference between different loss peaks and lead to excessive core energy loss; while too large a channel circle diameter will enhance the constraints on the core modes, resulting in the core mode energy being confined to the core region, making it difficult for the metal-excited equipartitioned excitations to couple effectively with the core energy [52]. The loss peak at d2 = 1.8 μm is the largest and is sharp, the half-height full width is the smallest, and the degree of coupling between the SPP mode and the core mode is the strongest.

### 3.3. Distance Between Channel Circles

**Figure 5:**
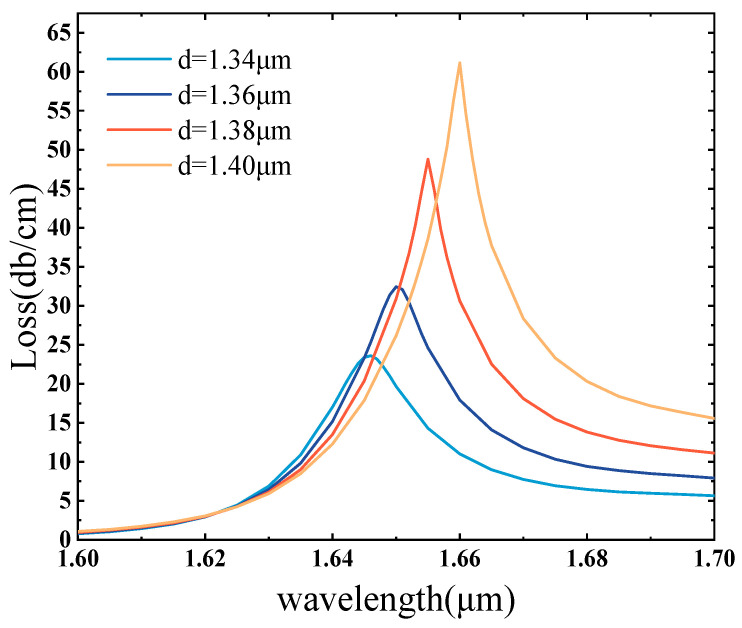
Variation in loss at 1.41 refractive index with inter-channel circumferential distance d of 1.34 μm, 1.36 μm, 1.38 μm, and 1.40 μm, respectively.

A suitable channel circle spacing can increase the touch area between the optical fiber and the surrounding medium, making it easier for the sensor to sense small refractive index changes. In this paper, the distance A between the two channel circles is varied with a gradient of 0.02 μm, and the distance between the two channel circles is expressed as 2d during the simulation. From Figure 5, it is apparent that the position of the resonance peak is gradually red shifted when d is adjusted in the interval spanning from 1.34 μm to 1.40 μm. This is because the SPP mode’s effective refractive index increases with d, causing a shift of the coupling wavelength towards longer wavelengths, while the loss value gradually increases [53,54]. In other words, as the channel circle distance increases, the constraint of the optical signal in the fiber core decreases, resulting in more optical energy leakage into the cladding region, and therefore, the pitch increases and the loss increases [55]. The resonant wavelength is 1.646 μm, 1.65 μm, 1.655 μm, and 1.66 μm loss increased from 23.61 dB/cm to 61.15 dB/cm. From Figure 5, it can be seen that when d = 1.34 μm and 1.36 μm, the two circles are too close to each other, the leakage window of the formation of the air holes is larger, and the perfect loss peak cannot be obtained. When d = 1.40 μm, the loss peak attains its maximum value, and the loss peak exhibits greater sharpness, and the coupling achieves the best effect. In the meantime, considering the peak factor and the structural limitations on the channel circle spacing, the channel circle spacing of d = 1.4 μm, i.e., A = 2.8 μm, is selected.

### 3.4. Distance of Fiber Core from D-Section H

**Figure 6:**
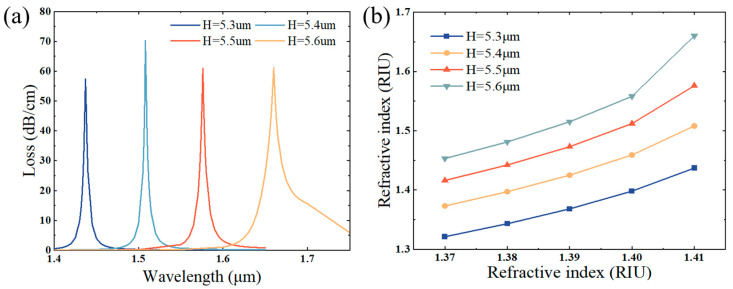
Loss variation at refractive index of 1.41, (**a**) core distance from the cross-section H = 5.3 μm, H = 5.4 μm, H = 5.5 μm, and H = 5.6 μm, and (**b**) effect of H on refractive index sensing sensitivity on the basis of the spr effect.

Figure 6a shows the fiber core’s loss spectral characteristics as the distance H from the D-section varies. Taking 0.1 μm as a gradient, as H increases from 5.4 μm to 5.6 μm, the resonant wavelengths are 1.437 μm, 1.508 μm, 1.576 μm, and 1.66 μm in order, and the losses are 57.35 dB/cm, 70.21 dB/cm, 60.95 dB/cm, and 61.15 dB/cm, which is manifest that the peak loss does not change much, but the resonance wavelength is gradually red shifted, mainly due to the larger H, which contributes to an increase in the effective area of the metal, and the effective refractive index of the real part of the surface equipartitioned excitation mode is gradually risen [56]. Figure 6b indicates the relationship curves between the coupling wavelength and the liquid’s refractive index to be measured for the PCF refractive index sensing based on the directional coupling effect under different H. The slopes of the curves in the figure reflect the sensitivity of this refraction index sensing system. The resonance wavelength and refractive index basically emerge a linear relationship, and the refractive index varies from 137 to 1.41, having a sensitivity of 2900 nm/RIU at H = 5.3 μm, 3375 nm/RIU at H = 5.4 μm, 4000 nm/RIU at H = 5.5 μm, and 5175 nm/RIU at H = 5.6 μm. It is observed that the greater the magnitude of H, the higher the sensitivity of refractive index sensing based on directional coupling, so although H = 5.4 μm makes the resonance peak sharper and the peak value reaches its maximum, with the sensor sensitivity requirements as the first priority, the comprehensive consideration of this paper adopts the H = 5.6 μm as the best parameter.

### 3.5. Analysing Performance by Changing Refractive Index

**Figure 7:**
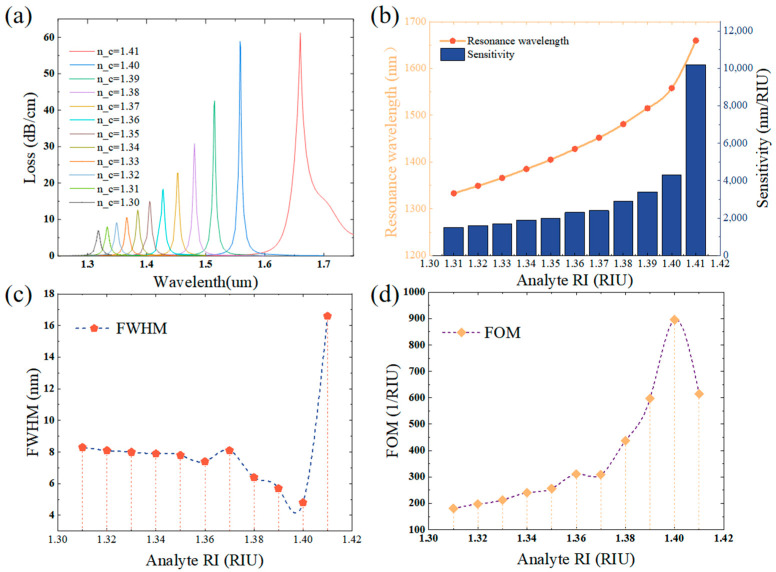
(**a**) Loss spectra with RI of 1.26~1.42 for t = 30 nm, d2 = 1.8 μm, d = 1.40 μm, and H = 5.6 μm, and (**b**–**d**) variation in sensitivity and resonance wavelength, FOM, and FWHM with refractive index for t = 30 nm, d2 = 1.8 μm, d = 1.40 μm, and H = 5.6 μm.

After optimizing the parameters of the proposed sensor, a simulation study of the sensor is carried out using the best parameters. In this section, the refractive index detection range of the proposed sensor is characterized and the results of the simulation study show that the sensor can operate in the refractive index range of 1.30–1.41, while the changes in the performance parameters of the sensor due to changes in the refractive index of the analyte are investigated [57,58]. Figure 7a demonstrates the response characteristics of the presented sensor to the analyte material within the refractive index span from 1.30 to 1.41. The alteration in the refractive index of the analyte to be tested causes a red shift in the coupling wavelength of the loss spectrum, and the coupling wavelength undergoes a transformation from 1.318 μm to 1.66 μm. The results of the modeling study indicate that the sensor has a good separation of the resonance peaks, which fully reflects its excellent resolution and sensitivity, which is in line with the experimental expectations and enables it to be applied to the detection of bioanalytes. Figure 7b illuminates the characteristic curves depicting the coupling wavelength as a function of the refractive index of the analyte, along with the sensor’s sensitivity for the respective refractive index values. Within the refractive index interval of 1.40 to 1.41, the sensor exhibits the largest resonance peak shift, and the peak value of its sensitivity reaches 10,200 nm/RIU, which is a data that fully confirms the outstanding refractive index detection capability of the sensor in the near-infrared wavelength band [59]. Figure 7c,d illuminates the FWHM versus the quality factor (FOM) of this sensor for the object under measurement with varying refractive indices. A high FOM is obtained when the sensor has superior sensitivity and a low value of the FWHM of the loss spectrum [60]. The simulation results of the COMSOL 6.0 software indicate that as the RI of the analyte rises, the sensor’s sensitivity enhances, and the response characteristics of the sensor display a nonlinear tendency. The smallest FWHM corresponds to a refractive index of 1.34 with a value of 4.8 nm, and the peak figure of merit (FOM) occurs at a refractive index of 1.34, with a numerical value of 895.83 (1/RIU).

**Figure 8:**
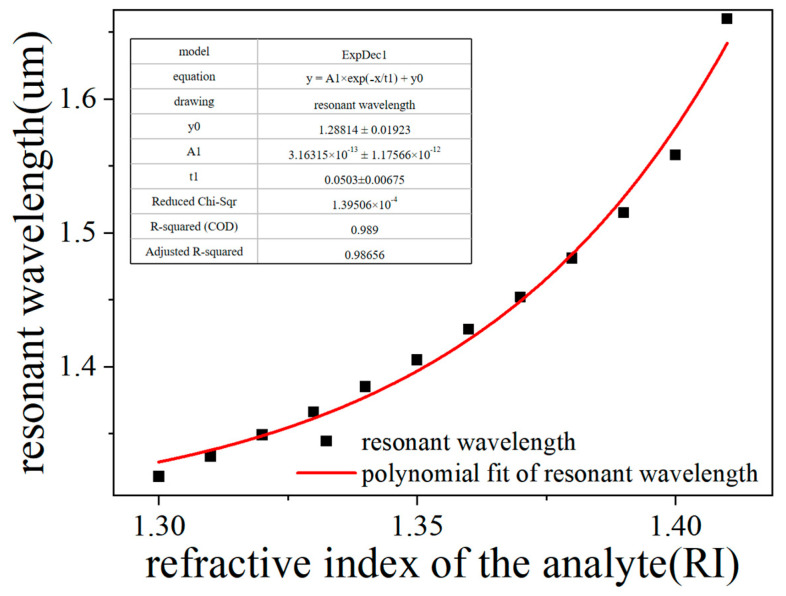
Fitted curve of resonance wavelength variation with analyte RI for different analyte Ris. (t1 > 0).

Figure 8 shows the fitted curve of the analyte’s index of refraction versus the resonance wavelength. The inset table indicates that the polynomial fitting curve has an adjusted R2 of 0.98656, indicating that the fit consistency is good. This step aims to combine the model with the real application scenario to enhance the practicality of the system by improving the fit. Specifically, the goodness-of-fit index reflects the capability of the model to characterize the sensor data, and the higher its value, the more accurately the model has the capacity to capture the characteristics of the sensor signals so that the sensor information can be extracted and processed more efficiently in practical applications [61,62,63]. This optimization not only enhances the reliability of data acquisition, but also provides higher-quality basic data support for subsequent data analysis and decision-making.

## 4. Performances

In this section, a software simulation sensor is used to detect glucose concentration in blood to investigate the sensor performance.

The concentration of glucose in the blood, often referred to blood sugar concentration, is an important indicator of the amount of glucose in the human body. As glucose concentration increases, the refractive index of the glucose solution increases accordingly. The sensor developed in the research possesses the merits of high sensitivity, anti-interference ability, portability, real-time monitoring, and micro-sample demand, which is suitable for detecting the blood glucose concentration. Refractive index data corresponding to different glucose concentrations in blood samples are crucial when modeling surface plasmon resonance properties, and these data can be measured with high accuracy by optical coherence tomography [64]. Glucose concentrations in blood of 50 mg/dL, 75 mg/dL, 100 mg/dL, 125 mg/dL, 150 mg/dL, and 175 mg/dL correspond to refractive indices of 1.352, 1.365, 1.375, 1.382, 1.394, and 1.405 [65]. Blood samples with different glucose concentrations are sequentially passed through the detection channel so that the analytes in the blood interact with the plasma wave to achieve accurate detection of the target analytes. In the simulation process, we use the above structurally adjusted sensor model, set the refractive index of glucose at different concentrations, and study its loss response characteristics sequentially. Additionally, enzyme immobilization techniques are employed to enhance stability, an approach that has been extensively utilized in biosensors [66]. Theoretically, after the designed sensor is prepared, the light source generates continuous incident light, which is then detected and analyzed by the sensing fiber. The detection information is encoded into the optical signal and transmitted through the fiber. The optical signal is subsequently received by the spectral analyzer, and the sensor’s detected information is displayed after data processing by the electronic computer [67].

**Figure 9:**
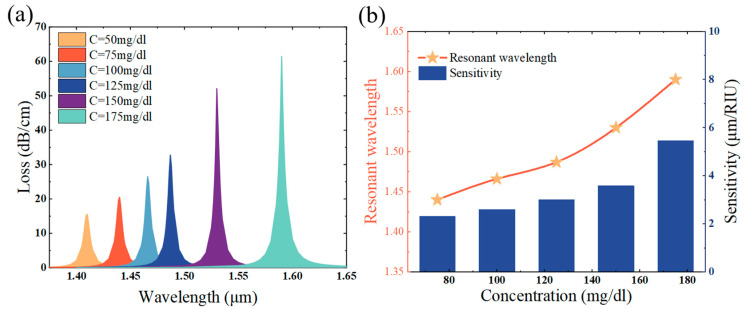
(**a**) Change in loss value of blood glucose concentration from 50 mg/dL to 175 mg/dL at t = 30 nm, d2 = 1.8 um, d = 1.40 um, and H = 5.6 um; (**b**) change in sensitivity and resonance wavelength of blood glucose concentration from 50 mg/dL to 175 mg/dL.

Figure 9a shows that with the increase in glucose concentration, the resonance wavelength of the loss peak is progressively moved towards the red end of the spectrum, and the coupling wavelength is gradually changed from 1.44 μm to 1.59 μm, and the magnitude of the loss peak gradually increases [68,69,70,71]. Figure 9b shows the relationship between the resonance wavelengths for different glucose concentrations, while the bar graph indicates that the sensitivity of the sensor to refractive index changes corresponding to different concentrations, from 75 mg/mL to 175 mg/mL, the refractive index sensitivity is 3750 nm/RIU, and the highest refractive index sensitivity is 5455 nm/RIU. The corresponding concentration sensitivities can be calculated by the following equation [72]:

(7) $$S_{\lambda }(\mathrm{nm}/\mathrm{mg}/\mathrm{dL})=\Delta \lambda_{R}/\Delta C.$$

The concentration sensitivity is 1.5 nm/mg/dL within the concentration variation of 75 mg/dL–175 mg/dL, and the highest concentration sensitivity is 2.4 nm/mg/dL. The PCF-SPR sensor put forward in this article for measuring glucose concentration in blood demonstrates superior sensitivity performance when compared to the previous sensors of this type, as shown the Table 2 [73,74,75,76], and it is expected to be put into use in the medical field.

## 5. Conclusions

Within this study, a PCF-SPR sensor that can be used for biosensing is designed and developed utilizing the finite element technique utilized through COMSOL 6.0 simulation software, and at the same time, its structural parameters are optimized; for example, the effects of the metal thickness, the channel circle distance, the dimension of the channel circle, and the dimension of the fiber core distance from the cross-section on the sensor are discussed so that the sensor has the optimal structure, sensitivity, and sensing effect. Then the loss response of the sensor within the refractive index range of 1.30–1.41 is discussed, and the results show that this sensor exhibits a good resonance peak separation. In the refractive index interval of 1.40 to 1.41, the sensor exhibited the largest resonance peak shift with the highest sensitivity of 10,200 nm/RIU, the smallest FWHM corresponded to a value of 4.8 nm at a refractive index of 1.34, and the maximum FOM corresponded to a refractive index of 1.34 with a value of 895.83 (1/RIU). Additionally, to measure the concentration of glucose in blood as an example, the response of the sensor at various concentrations was examined, and the results indicate that with the concentration of glucose in the range of 75 mg/dL–175 mg/dL, the sensor exhibits a refractive index sensitivity of 3750 nm/RIU. Among all the measured sensitivities, the highest refractive index sensitivity stands at 5455 nm/RIU, suggesting that under certain conditions, the sensor can offer enhanced sensitivity for precise refractive index measurements. By inference, the sensor can also be measured in other biological fluids used in the field of biosensing and medical research, which is exploitable.

## Figures and Tables

**Table 1:** Initial Setting Parameters.

Notation	Parametric	Numerical Value
R0	PCF radius	11 μm
d1	Diameter of No. 1 air hole	1.4 μm
d2	Diameter of air holes No. 2	1.8 μm
d3	Hole 3 diameter	0.8 μm
t	Metal film thickness	30 nm
A	Distance between two channel circles	2.8 μm
H	Core distance from D-section	5.6 μm
M	PML layer thickness	1 μm

**Table 2:** The designed sensors are compared with previously published sensor models.

Maximum Sensitivity (nm/RIU)	Detection Range	Bibliography
225	1.34–1.44	[73]
750	1.330–1.401	[74]
400	1.355–1.341	[75]
1278	1.50–1.60	[76]
5455	1.352–1.405	We propose a glucose sensor

## Data Availability

Publicly available datasets were analyzed in this study. This data can be found here: [https://www.lumerical.com/ (accessed on 1 January 2020)].
